# Developing creative and research skills through an open and interprofessional inquiry-based learning course

**DOI:** 10.1186/s12909-019-1563-5

**Published:** 2019-05-08

**Authors:** Gemma Rodríguez, Nora Pérez, Gemma Núñez, Josep-E. Baños, Mar Carrió

**Affiliations:** Research Group in Health Sciences Education, Department of Experimental and Health Sciences, Universitat Pompeu Fabra, Dr. Aiguader 88, 08003 Barcelona, Spain

**Keywords:** Research skills, Inquiry-based learning, Creative thinking, Active learning, Higher order skills

## Abstract

**Background:**

Biomedicine needs innovative professionals. Inquiry-based learning (IBL) aims to develop higher order thinking skills, such as creativity and research. Stimulatory techniques and interprofessional education, which requires students from different fields to collaborate, also enhances creativity. In this study, the effectiveness of an interprofessional IBL course that introduces a creativity workshop based on stimulatory techniques to develop creative and research skills is examined.

**Methods:**

529 undergraduate human biology and medical students performed the interprofessional IBL course, 198 with the creativity workshop and 331 without. Students’ perceptions of learning processes and outcomes were assessed in surveys and focus groups by the authors of this study. As well, the final learning results from both groups of students were analyzed by the teachers of the course and the researchers.

**Results:**

The results show that the open IBL approach promoted the development of these skills, interprofessionality acted as a creativity enhancer and stimulatory techniques contributed to improve the learning outcomes.

**Conclusions:**

This study provides insight into how open interprofessional IBL fosters acquisition of complex skills and knowledge, pointing out the benefits and limitations of this approach in health sciences studies.

## Background

Changing social demands require knowledge and skills that can be applied across disciplines. Professionals who can analyze issues, think critically, solve problems, communicate effectively, and take leadership are essential to meeting new challenges in modern societies. In this context, what counts is not just the knowledge domain, but the capacity to think and learn, communicate, and collaborate [1]. Professionals trained in creative thinking, problem-solving, self-directed learning, team building, and other high level abilities are needed to address complex interdisciplinary challenges [1–3]. Therefore, higher education needs to emphasize these skills and introduce training in creative thinking so graduates can meet these challenges.

Creativity is key in research and innovation processes. Processes such as formulating new questions, deductive and inductive reasoning, and combining unrelated knowledge are closely linked to creative thinking [4]. Although creativity is often considered the ability to generate new and original ideas [5], it can also be conceived as the interaction between aptitude, process, and environment where individuals produce something new and useful in a social context [6]. Thus, creativity is a collaborative, social phenomenon requiring interaction and discussion [7, 8].

Generating scientific knowledge requires communication and interaction with other scientists; interdisciplinary and collaborative work are key [9]. Thus, creativity is intrinsically tied to the nature of science and scientific knowledge [10]. The creative process of science encompasses discovering new scientific problems, deriving hypotheses based on existing knowledge, designing new experiments, evaluating evidence, and verifying theories. This approach allows diverse routes to solutions with novel combinations of knowledge or techniques [11, 12]. Hu and Adey (2002) synthesized the literature about creativity and the nature of science to propose a scientific structure creativity model in which scientific creativity is a combination of the creative process, the characteristics of the creative person, and resulting products [11].

Promoting creativity should be a priority for education systems to meet the challenges of the twenty-first century [13]. Active learning strategies are more effective than traditional teaching in promoting creative thinking [4, 14]. Some studies demonstrate that pedagogical approaches based on interaction, inquiry, problem-solving, and interdisciplinarity in flexible and open environments foster students’ autonomy, responsibility for their own learning, cooperative work, and long-term knowledge retention [15], and might also promote the acquisition of creative thinking skills [14].

Interprofessional education (IPE) is defined as the occasion where learners from two or more different health sciences domains learn with, from and about each other to improve collaboration and health outcomes [16]. Approaches that encourage IPE where these members from different domains integrate knowledge from their disciplines and work collaboratively towards a common purpose, also enhance creativity [17]; as well as the interaction of multiple perspectives and potentially constructive conflicts can enhance and increase creativity [18].

Student-centered approaches, such as inquiry-based learning (IBL), are optimal for promoting creative thinking. IBL models the general investigative process that scientists use by simulating real investigations in which students acquire different skills to apply knowledge and find solutions to complex problems [19–21]. IBL enhances higher order skills, including self-reflection, critical thinking, the ability to undertake independent inquiry, and a sense of responsibility for learning, intellectual growth, and maturity [22, 23]. IBL can integrate research and teaching where students and teachers act as co-learners [23].

IBL assumes different forms depending on the nature of the inquiry, level of guidance during the process, learning priorities, and scale. IBL is classified as structured inquiry when teachers provide an issue or problem and outline for addressing it; as guided inquiry when teachers provide questions to stimulate inquiry but students are self-directed in exploring these questions; and as open inquiry when students formulate the questions and control the full inquiry cycle [23]. Open inquiry is more likely to foster creative skills. To foster creativity in IBL, teachers can use stimulatory techniques (e.g., brainstorming or checklists), problem-solving, or expert facilitation techniques [24]. Working with multidisciplinary groups can also enhance creativity through sharing of different viewpoints and experiences [24].

We hypothesized that an open-IBL approach that promotes interprofessionalism can enhance the development of creativity and research skills in undergraduate health sciences students. To test this hypothesis, we designed a course with the following elements: 1) an open-IBL project in which students freely decided what and how to investigate, 2) the promotion of interprofessionalism by allowing undergraduate students from different backgrounds to work together on projects reflecting common interests and 3) a workshop on stimulatory techniques to enhance the development of creative skills. So, the aim of this study is to evaluate the development of research and creativity skills in undergraduate biomedical students through this IBL approach and evaluate the impact of the creativity workshop. To assess this main goal, we analyzed students’ perceptions of their learning process and expected outcomes and the final projects produced by the students (i.e. the actual learning outcomes) were assessed.

## Methods

### Research context

Since 2011, a full and open-IBL course “Integrated Biomedicine” was offered to third-year students in the Bachelor of Human Biology and Bachelor of Medicine programs, respectively, enabling students from the two programs to work together to find creative solutions to challenging real problem (e.g., antibiotic resistance, microbiome and human health, human immunodeficiency virus and tuberculosis or cancer immunotherapy). In 2014, a creativity workshop, using stimulatory techniques was introduced in this course to increase the development of creativity.

### The open-IBL approach

The open-IBL model allowed students to conduct their research project in small groups with a teacher acting as a learning facilitator. The main objective of this course was to develop students’ creativity, research and collaborative skills. Intended learning outcomes were to be able (a) to define a relevant, original, and feasible research question; (b) to formulate a hypothesis based on background information; (c) to plan data collection and analysis to answer the research question; (d) to discuss the results and draw conclusions; and (e) to communicate the results through a written paper and an oral presentation. These research skills were developed while working in cooperative teams made up of students from different backgrounds.

The course lasted ten weeks. Groups comprised eight to ten students, half from each degree program, who worked with a tutor in a two-hour session each week. First, a broad ill-structured problem in biomedicine was presented through a short video (10–15 min) showing the key features of the scenario, followed by brainstorming. Afterwards, each group had to define a research question to address the proposed scenario. Students were free to choose any option, provided they could elaborate a feasible working hypothesis and suggest appropriate methods to obtain an answer (e.g., experiments in the school laboratories, consultations with researchers, big data analysis or using patients’ hospital data). During the development of the research project, tutors guided students through process and gave them feedback. At the end of the course, the students presented their results in a symposium.

### The creativity workshop

A four-hour creativity workshop was introduced in 2014 to provide tools to better develop creative skills in the context of this open-IBL course. This workshop aimed to prompt reflection about creativity and its importance in biomedical research, to introduce various creativity techniques and tools, and to provoke collaborative reflection on how these techniques could stimulate creative knowledge construction. Four groups of students and two trainers worked in the same classroom. The workshop had two sessions: the first to train students in generating new ideas and the second to assess and improve the ideas generated. The first session took place in the second week of the term, when students were still defining their research projects. The second session took place in the fifth week, when students had already defined their research proposals and were developing their projects. Table 1 summarizes the workshop plan.

**Table 1:** Creativity workshop plan. Identification and detailed description of the activities and its objectives in the Session 1 (Generation of new ideas) and Session 2 (Assessment and improvement of ideas) of the creativity workshop

Session 1. Generation of new ideas		
Activities	Objectives	Description
Debate about the role of creativity in biomedical professions	To identify students’ previous ideas and concepts on this issue.	Through a real-time questionnaire, students determine their beliefs about creativity in biomedical professions and then reflect collaboratively.
Brainstorming (post-it®)	To produce many new ideas in a short time for their research project proposals.	Participants have two minutes to think on a problem. Each must write at least one idea on a post-it®. Each post-it® is stuck on the wall. The group discusses all the ideas, categorizing and prioritizing them according to their usefulness for resolving the problem.
Heuristics ideation	To generate new concepts, ideas, products, or solutions connecting different concepts.	Participants write two lists, one containing motivational concepts or issues in the science field and the other including ideas from the brainstorming. The group must associate concepts from the two lists and generate new ideas.
Role storming	To generate ideas from different viewpoints so the research proposal can be analyzed from different approaches.	Participants choose an admirable or despicable personage and imagine what this person would think about their project. Afterwards, they analyze and discuss the emerged ideas.
Six hats De Bono [25]	To encourage the analysis of the project from multiple perspectives.	Each participant has a hat that symbolizes a way of thinking: emotion, creativity, optimism, information, control, or logic. Participants must answer all the questions related to their specific hat and the main problem.
Session 2. Assessment and improvement of ideas		
Activities	Objectives	Description
Strange object	To promote the use of analogies to change the reference framework where students look for solutions.	Participants write an analogy between a strange object from daily life and the project.
Ishikawa diagram [26]	To reorganize concepts and ideas linked to the project.	Concepts and ideas identified must be grouped into different categories connected to the problem or the project in a diagram.
SWOT	To analyze the project’s potential strengths, weaknesses, opportunities, and threats to find ideas to improve the project.	Participants reflect and complete the SWOT matrix.
SCAMPER [27]	To find new ideas to improve the product or the process developed during the project.	Participants must ask questions related to improving the project through Substituting, Combining, Adapting, Modifying, Putting to other purposes, Eliminating, and Replacing.
Logo design	To synthesize the main project idea and highlight its essence through symbolic language.	Participants must design a logo for their own project.

### Participants

This project was carried out during the 2014–2015 and 2015–2016 academic years. The students who performed this subject during 2014–2015 and 2015–2016 and performed the creativity workshop were compared to the three previous promotions (2011–2012, 2012–2013 and 2013–2014), which performed this subject without the creativity workshop. A total of 529 students participated in these courses (331 in the 2011–2014 promotions, and 198 in the 2014–2016). Students enrolled in all these academic years were asked to complete a survey about the course; 175 students accepted (49% of the students from the 2014–2015 and 2015–2016 cohorts and 26% of those from the previous cohorts). Of these, 101 were from the Bachelor of Human Biology program and 74 from the Bachelor of Medicine program; 127 participants were women and 48 were men. Moreover, a total of 32 students from 2014 to 15 and 2015–16 participated in focus groups. Table 2 shows participants’ main characteristics.

**Table 2:** Characteristics of students surveyed in the study

Cohort	Students (n)		Gender (M/F)	University entry examination scores^a^		Creativity workshop
	HB	M		HB	M	
2011–2012	57	21	19/59	8.1	8.7	
2012–2013				7.9	8.5	No
2013–2014				8.0	8.6	
2014–2015	46	51	29/68	8.2	8.4	Yes
2015–2016				8.3	8.8	

*HB* Bachelor in Human Biology program, *M* Bachelor in Medicine program

^a^The maximum possible university entry examination score was 10

### Data collection and instruments

This descriptive-evaluative research study used a combination of quantitative statistical techniques and qualitative content analysis methods to analyze data collected considering the objectives of the study. To test the hypothesis that an open-IBL approach that promotes interprofessionalism can enhance the development of creativity and research skills, four aspects were assessed: 1) Students’ perception of their development in research skills and creative thinking during the course 2) students’ learning experience (i.e. their opinions and personal experiences with the open-IBL course), 3) the impact of the implementation of the creativity workshop introduced in the cohorts 2014–2015 and 2015–2016 to better promote the development of creativity skills and 4) evidence of development of research and creative thinking skills.

To this end, various data collection instruments were used regarding these aspects:

#### Students’ perception of their development in research skills, creative thinking and learning experience

- Questionnaire: at the end of the course, students completed an anonymous questionnaire with several closed-ended questions and an open section for general comments. Participants rated from 0 (not at all) to 10 (extremely) the following items:
  - Development of research skills: Improvement of five skills: identifying relevant research questions, formulating hypotheses, designing research projects, collecting and analyzing data, and reaching conclusions and contributions. Mean scores to five questions were used for further analyses.
  - Creativity of the research project: Students rated the research project’s originality, value, and usefulness. Mean scores to three questions were used for further analyses.
  - Creativity enhancers: Students rated their agreement with the statements: “An open scenario promotes creativity”, “Cooperative and interprofessional work fosters the development of creativity” and “The IBL process fosters the development of creativity”.
  - Working in interprofessional teams: Students answered whether this experience enhanced their interest in working in interprofessional teams.
  - Satisfaction with the course.
  - Usefulness of the course.

#### Implementation of the creativity workshop

- Questionnaire: at the end of the course, students completed an anonymous questionnaire with several closed-ended questions and an open section for general comments. Participants rated from 0 (not at all) to 10 (extremely) the following items:
  - General satisfaction with the creativity workshop.
  - Utility of the different activities and quality of the materials used.
  - The learning environment.
  - The trainers’ support.
  - How the workshop had contributed to the project.
- Focus groups: During 2014–15 and 2015–16, 1-h focus group sessions (two with Human Biology students and two with Medicine students) were performed to collect information about the IBL approach, interprofessionalism, the role of the tutors and the creativity workshop.
- Field notes: Methodological, descriptive, and personal field notes were collected in a total of 12 creativity workshop sessions (3 for each creativity workshop session during the two academic years).
- Academic results: the final grades of the students who did the creativity workshop were compared with those of the students who did not.

#### Evidence of development of research and creative thinking skills

- Academic results: students’ final grades were analyzed.
- Research projects analysis: The 25 research projects done during the academic years 2014–2015 and 2015–2016, that performed the creativity workshop, were analyzed for creativity.

### Data analysis

SPSS software was used for quantitative analyses. To analyze correlations between quantitative variables, we used Pearson’s *r* because all the variables had a linear relation between them and a normal distribution. The two variables that didn’t have a normal distribution were transformed through a square root and adjusted to a normal distribution to perform Pearson’s *r.* To determine whether values of variables differed between gender and between students in the Medicine and Human Biology programs, we used Student’s t-test as appropriate because the normal distribution of the variables [28]. To analyze the results of the focus groups, students’ comments, and field notes, we used Atlas.ti software for qualitative content analysis within a constructivist paradigm. Codes and categories that emerged during the analysis were refined after multiple iterations of content coding [29–31].

Furthermore, intended learning outcomes were assessed by different activities during the course: 1) students’ participation, assessed by tutors and team peers, counted for 30% of the final grade; 2) two mid-reports, assessed by tutors, counted for 20% of the final grade; 3) students’ final scientific papers, assessed by three teachers, counted for 25% of the final grade, and 4) students’ final oral presentations at the symposium, assessed by three experts on the topic, counted for 25% of the final grade.

Finally, to assess the scientific creativity of students’ research projects, we used Hu and Adey’s model [11], taking into account the products developed (technical product, advance in science knowledge, understanding of scientific phenomena, and scientific problem solving) and their level of creativity (calculated by the mean of *originality*, defined as an answer that is rare, which occurs occasionally in a given population, the *value*, defined as importance in a given context, and *usefulness*, defined as the aptitude to satisfy a need) [11, 32].

## Results

### Students’ perception of the development of research skills and creative thinking

Tables 3 and 4 report the main results of descriptive and correlational analyses to assess students’ perception of the development of research skills and creativity during the course and their opinion on how the variables “open scenario”, “cooperative work”, and “inquiry process”, named creative enhancers, fosters the development of creativity.

**Table 3:** Descriptive statistics using scores from 1 = strongly disagree to 10 = strongly agree to assess the students’ perception of the development of creativity and research skills through the inquiry-based learning approach and the role of the creativity enhancers (*n* = 175)

			Creativity enhancers		
	Research skills	Creativity	Open scenario	Cooperative work	Inquiry process
Mean	7.37	7.68	8.51	8.08	7.90
Median	7.50	8.00	9.00	8.00	8.00
SD	1.44	1.81	1.52	1.97	1.79
Variance	2.08	3.29	2.32	3.88	3.20

**Table 4:** Pearson’s correlation analyses between the students’ perception of having developed creativity, research skills and the role of creativity enhancers in the open IBL course

			Creativity enhancers		
	Creativity	Research skills	Open scenario	Cooperative work	Inquiry process
Creativity	1	–	–	–	–
Research skills	0.64^a^	1	–	–	–
Open scenario	0.47^a^	0.41^a^	1	–	–
Cooperative work	0.43^a^	0.50^a^	0.55^a^	1	–
Inquiry process	0.54^a^	0.66^a^	0.52^a^	0.69^a^	1

^a^Statistical significance at 0.01 (bilateral) (*n* = 175)

Students’ ratings were high for all items, especially for research skills and creativity. Students considered that creativity enhancers such as the “open scenario”, “cooperative work”, and “inquiry process” foster the development of creativity. No significant differences were found between scores of students from the two degree programs, neither between men and women. All correlations between variables were significant; the strongest correlations were between “cooperative work” and “inquiry process” (r = 0.69), “research skills” and “inquiry process” (r = 0.66), and “research skills” and “creativity” (r = 0.64).

Furthermore, no significant differences were found on the perception of creativity development between the promotions that did not perform the creativity workshop (2011–2012, 2012–2013 and 2013–2014) and the promotions that did (2014–2015 and 2015–2016).

Table 5 reports the qualitative analysis of the development of research skills and creative thinking based on students’ comments, focus groups, and field notes. Despite this study has obtained more data from the cohorts that performed the creativity workshop, and consequently, more information, the comments from the two different cohorts are in the same vein as the results presented in Table 5.

**Table 5:** Qualitative results of the development of research skills and creative thinking, during the learning process and as a learning result, obtained through the comments of the students that performed the open IBL course (*n* = 175), and the focus groups (*n* = 32)

Category	Subcategory	Subcategory	Findings	Quotes
Learning process	Creative process of knowledge construction	Open scenario	An open scenario stimulates creative thinking: students seek original ideas to differentiate themselves from other groups and are free to decide what to do and how to do it. This results in new ideas, integrating different fields and perspectives.	*Open problems make you differentiate yourself from the other groups, be original. If they were closed, you would probably be less original.* *If we can choose and decide, it is easier to have more options and to create.* *You realize all the ways you can focus on a problem—all the fields, topics, and methodologies to use.*
		Cooperative work	Working with peers from different fields makes students integrate each other’s perspective to reach a group consensus, makes a more complete project, and adds value.	*If we can understand each other, we can touch more fields and do a better project.* *Others’ strengths and perspectives compensate for our shortcomings.*
		Inquiry	Inquiry cycle makes students analyze the situation and propose new ideas, apply knowledge, and seek alternative solutions.	*In this process, when we have a problem, we must seek alternative solutions, decide which is best, and think.*
		Limitations	Openness makes students choose creative projects that are difficult to realize. Expectations do not correspond to time. Friction between peers and difficulties during the project can limit creativity.	*The topic was too open and at first we didn’t know how to focus the project.* *We must be careful with expectations; we thought we could do a project and then we realized we had insufficient time. The funding was inadequate for our project design.*
Learning outcomes	Skills development	Transversal skills	Oral and written communication, critical search for information, and self-learning skills were developed.	*We all contributed to writing.* *We had to explain to peers what we had done and found every week.* *We had to decide alone what we wanted to do and how we had to do it.*
		Research skills	Students gained experience in the designing laboratory experiments, searching for protocols, planning interventions, analyzing problems, seeking solutions, and evaluating contributions.	*If you want to do a research project, you need to do library research but also plan an experiment, develop it, and analyze the results.* *For the first time we had to plan a project and all this entails*.
		Critical thinking	Discussing problems with peers gave students a critical view of the possibilities, limitations, and improvements of their research.	*We were able to develop a critical view, reflecting on and discussing our project.* *We evaluated what we had done and said what we could improve.*
	Scientific product	Scientific product	Most projects were original, society-related, and integrated different fields and perspectives.	*Some people did educational projects, others basic research. One group designed a communication proposal. Others more epidemiological projects...*

### Students’ learning experience

Students’ IBL-based learning experience was assessed through quantitative and qualitative methods considering their perception of having developed creativity and research skills as well as their valuation on the usefulness of the course for their training and the general satisfaction with the course. No significant differences were found between men and women. Tables 6 and 7 show the descriptive statistics and correlation analysis.

**Table 6:** Descriptive statistics of the assessment of the students’ learning experience regarding Satisfaction and Usefulness with the IBL approach (*n* = 175)

	Satisfaction	Usefulness
Mean	7.48	7.39
Median	8.00	8.00
SD	2.05	2.10
Variance	4.22	4.40

**Table 7:** Pearson’s correlation analyses between the students’ learning experience (Satisfaction and Usefulness variables) with the students’ pereception of having developed creativity and research skills (Creativity and Research skills variables)

	Creativity	Research skills	Satisfaction	Usefulness
Satisfaction	0.61^a^	0.69^a^	1	–
Usefulness	0.57^a^	0.71^a^	0.84^a^	1

^a^Statistical significance at 0.01 (bilateral) (*n* = 175)

Students rated their satisfaction and the usefulness of the course highly. Human Biology students’ ratings were higher than those of Medicine students (7.96 vs. 6.77 for satisfaction, *p* = 1.38 × 10^−4^, and 7.98 vs. 6.51 for usefulness, *p* = 2.94 × 10^− 6^). Non significant differences were found between the promotions that did not performed the creativity workshop (2011–2012, 2012–2013 and 2013–2014) and the promotions that did (2014–2015, 2015–2016) in terms of satisfaction and usefulness. When asked if they considered working collaboratively in interprofessional groups interesting, 87% answered affirmatively and 13% negatively.

All correlations between items were statistically significant. Strong correlations were seen between “satisfaction” and “usefulness” (r = 0.84), “usefulness” and “research skills” (r = 0.71), “satisfaction” and “research skills” (r=0.69), and “satisfaction” and “creativity” (r=0.61). A lower correlation was found between “creativity” and “usefulness” (r = 0.57).

Table 8 reports the qualitative analysis of students’ learning experience based on students’ comments and the focus groups results. The comments analyzed from the two different cohorts are along the same lines as the results exposed in Table 8.

**Table 8:** Qualitative results of students’ opinions on the open-IBL experience regarding satisfaction, usefulness, interprofessionalsim, the tutors of the subject, evaluation, experienced emotions and limitations. Results obtained from the students’ comments (*n* = 175) and the focus groups (*n* = 32)

Category	Findings	Cites
Satisfaction	Students were satisfied with their project and with the methodology, perceiving that IBL promotes long-term knowledge retention.	*What you learn with open-IBL you retain longer.*
Usefulness	Students considered it useful for their future: how to work in a lab and do field research; also useful for final bachelor project.	*It prepares you for the final bachelor project, but also for research. You are more prepared for the professional world.*
Inter-professionalism	Students considered interprofessionalism a positive experience: They learned to work cooperatively, with ideas from different fields, and consider ways of working useful for the project and future.	*Multidisciplinary teams provide us with different views and help us work cooperatively with peers from other fields.*
Tutors	Different kinds of tutors participated. Students considered the ideal tutor should have previous experience, guide, and give freedom—not just evaluate.	*I think that the ideal tutor is a balance between guidance and freedom and awareness of real possibilities.*
Evaluation	Students appreciated the assessment and expert committee assessing the projects. However, they saw some limitations, such as the bias of the experts assessing interdisciplinary projects.	*The formative assessment was useful to keep up to date, but the committee assessed the projects depending on the field.*
Emotions	Positive: motivating, interesting, involvement, competition, creativity.	*You can choose the topic, so we look for an interesting project that motivated and engaged us. We had lot of work. It put pressure on me.*
	Negative: confusion and difficulties during the process, anxiety for amount of work.	
Limitations	Organizational issues, timing of the subject, tutors and evaluation	*Ten weeks is not enough time to carry out the project. It was difficult to meet the deadline.*

### Implementation of creativity workshop

**Fig. 1:**
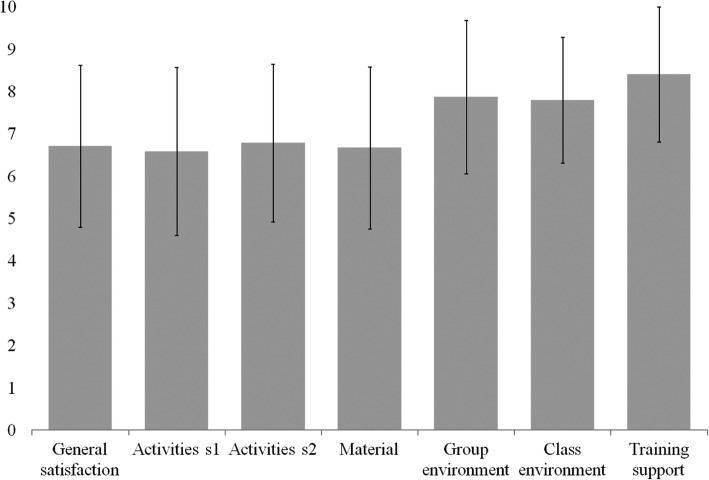
Quantitative assessment of the creativity workshop. Data are expressed as mean and SD of each variable (*n* = 97)

We used quantitative and qualitative methods to analyze items related to general satisfaction with the creativity workshop and with the activities and atmosphere in the workshop (Fig. 1).

Students’ scores for variables related to general satisfaction, activities, and material used in the creativity workshop were moderately high. Students rated “training support”, “group environment”, and “class environment” highly. No significant differences were found between students from the two degree programs. Furthermore, 68% of students consider that this creativity workshop helped improve the creativity of their research projects.

Table 9 reports the results of the qualitative analysis of the creativity workshop based on students’ comments, focus groups, and field notes.

**Table 9:** Results of the qualitative analysis of the implementation of the creativity workshop regarding strengths and weaknesses of the implementation through the students’ comments (*n* = 97) and the focus groups (*n* = 32)

Category	Subcategory	Findings	Cites
Creativity workshop	Strengths	The workshop was useful for choosing and delimiting project topics and for group cohesion. Students felt that these sessions promoted more freedom than tutored sessions. The techniques considered most useful were idea generation and evaluation techniques (Brainstorming, SWOT).	*New ideas emerged during the workshop.* *The free environment helped unite the group.* *Brainstorming was useful for organizing our ideas.*
	Weaknesses	Timing of the sessions, need for a tutor in each group, too many techniques.	*I think that fewer techniques would be better for this kind of workshop.*

### Evidence of development of research and creative thinking skills

#### Learning outcomes results

Table 10 shows students’ final grades (including all the assessment activities) and symposium grades.

**Table 10:** Summary of students’ grades (final grade and symposium grade) during the study period, by academic year (2011–2016) (*n* = 529)

		Final grade (maximum = 10)		Symposium grade (maximum = 10)	
Academic year	N	Mean	SD	Mean	SD
2011–2012	111	8.6	0.54	8.8	0.43
2012–2013	108	8.4	0.53	8.2	1.0
2013–2014	108	8.6	0.45	8.7	0.37
2014–2015	109	8.8	0.47	8.9	0.26
2015–2016	92	8.7	0.52	9.0	0.43

Students’ final grades were very high in all the academic years, indicating that most students clearly acquired the required research skills; moreover, the low SD suggests learning results were homogenous. Both tutors involved in the course and the expert committee composed of physicians and researchers qualified the students’ final projects as very good.

Significant differences were found between the final grades obtained by the promotions that performed the creativity workshop (2014–2015 and 2015–2016) and the promotions that did not (2011–2012, 2012–2013 and 2013–2014). Final grades were higher in the promotions that performed the creativity workshop than the promotions that did not (8.75 vs. 8.55, *p* = 1.17 × 10^4^).

#### Creativity development assessment through the evaluation of the students’ productions

**Fig. 2:**
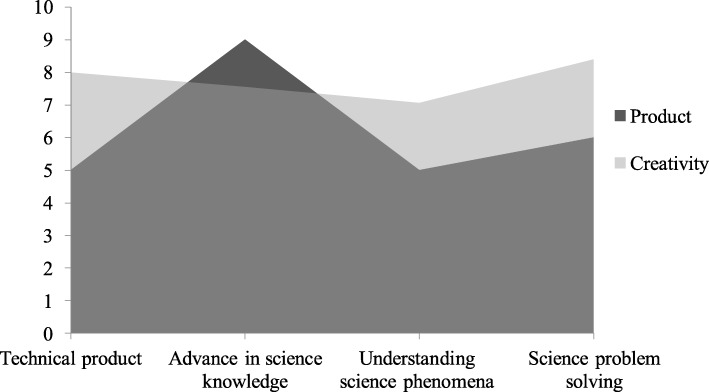
Assessment of the creativity of students’ research products (*n* = 25). “Creativity” represents the mean of “originality”, “usefulness”, and “value” of each research project

Figure 2 shows an assessment of the creativity of students’ projects, categorized according to the type of creative product.

The most common type of creative product aimed to “advance in science knowledge” (*n* = 9). One example of this kind of product is a project that determined variations in the vaginal microbiota associated with the use of tampons versus menstrual cups and the possible association of these methods with increased susceptibility to genitourinary diseases. The second most common type aimed to “solve a science problem” (*n* = 6). One example is a project that studied the formation of biofilms in vitro to test a disaggregating drug, which also potentiates the antimicrobial effect of antibiotics, to solve the problem of high antibiotic resistance in bacteria that colonize medical devices. The remaining creative product types aimed to “design a technological product” (*n* = 5) or “understand a science phenomenon” (n = 5). One example of the former is a research project conceiving a non-toxic probiotic product that could alter the human skin microbiome to make it less attractive to mosquitoes that transmit disease; one example of the latter is a project that studied the high prevalence of resistance to antidepressants and its relation with the intestinal microbiome and inflammatory processes.

Figure 2 also shows that all the scientific products developed were rated high for creativity. The products types focused on science problem-solving and designing technical products were the most creative (8.4 and 8.0, respectively). However, other products types such as advances in science knowledge or understanding science phenomena also scored high in creativity (7.6 and 7.1, respectively).

## Discussion

### How have creativity and research skills been developed?

This study examines the development of research skills and creative thinking through an open-IBL course. Students perceived that they had acquired these skills after the course. Furthermore, the development of research skills correlated strongly with the students’ thought of having developed a creative project.

As Justice et al. (2009) explained, IBL can enhance learning outcomes, such as the development of higher order skills (creativity, critical thinking, and research skills), as well as strengthen the teaching-research nexus [1, 33]. Domain-knowledge and skills are major components of creativity [34], and scientific exploration and activities such as defining scientific problems, formulating hypotheses, designing research plans, evaluating evidence, and verifying further theories are considered key for developing scientific creativity [12]. Thus, investigating different aspects of a problem develops creativity [4].

Our results confirm that based on students’ perception an open problem, cooperative and interprofessional work, and the inquiry process itself can enhance creativity. Quantitative analyses found strong and moderate correlations between students’ perceptions of the development of research skills and creativity and the open-IBL approach; and qualitative analyses of students’ comments and focus groups reinforce these findings. Students considered that open-IBL stimulated creative thinking: freedom to decide what to do and how to do it fostered original new ideas promoted by the integration of different fields and perspectives, and it strengthened students’ ability to define research proposals. These results agree with previous studies that identified open- and discovery-oriented IBL as the IBL models that best promote higher-order learning outcomes, including the definition of scientific problems, design of an appropriate method of study, and capacity to do research [23, 35]. Students also pointed to the important role of cooperation among peers with different backgrounds in the creative process of knowledge construction (mean score = 8.1). These results support previous research findings that collaboration, exchange of ideas, and different perspectives enhance creative thinking and the development of research skills [19]. Moreover, according to Zhou (2015), in collaborative contexts participants build on each other’s ideas through critical and constructive negotiations to each other’s suggestions to reach an understanding that is initially unavailable to any individual participant [36]. In our study, 87% of the students from the two degree programs found working together interesting; Oandasan and Reeves (2009) explained that interprofessional education enhances the development of creative thinking, skills development, and construction of collective knowledge [17, 18].

The qualitative results obtained in the analysis of the open-IBL implementation and the creativity workshop suggest that workshop sessions, where students felt free to express their ideas, could have been more conducive to the development of creative projects than the tutorial sessions. Freedom and flexibility in situations where students need to apply knowledge and solve problems are key for the development of scientific creativity [37], although, as our qualitative assessment shows, the development of creative thinking and research skills can be limited by tensions between peers, openness and time, or difficulties during the research project. The development of scientific creativity requires tolerance and safe, democratic environments [38].

Despite non significant differences were found in perception of creativity development between the students who performed the creativity workshop and the students who did not, neither in satisfaction and usefulness of the inquiry approach; the quantitative and qualitative data supports the idea that most students considered that the creativity workshop contributed to and had an impact on the development of creativity in their projects. The creativity workshop, introduced in the academic year 2014–2015, has been useful and has allowed us to compare the different cohorts. In fact, the promotions 2014–2015 and 2015–2016, which performed the workshop, perceived higher values on the products’ creativity and their finals grades were significantly higher, compared with the ones that did not performed the workshop. They reported that this workshop promoted group cohesion and helped them define the research proposal. Students considered brainstorming, heuristics, and analogies or visual diagrams to analyze different elements of the project useful, but also pointed out that time constraints meant that some techniques were used only superficially and that employing fewer techniques might be more useful. These results support the theory that interactive group sessions promote creativity by encouraging participants to develop and share ideas and connections, stimulating idea generation and evaluation, promoting alternative thinking, unexpected connections, parallel group thinking, and problem solving [24]. Previous research and our results show that these techniques stimulate creative thinking, but require time to be more effective. Thus, we recommend introducing some of these techniques in open-IBL courses.

Our analysis of students’ projects also confirmed the development of creativity. As Hu and Adey (2002) explain, scientific creativity can be assessed by the type of product generated, its originality, value and usefulness. Creative skills were evident in students’ solutions to science problems (i.e., scientific products) and the traits that define creativity manifest in the high scores for students’ products [11].

Finally, students’ grades demonstrate that the intended learning outcomes were clearly achieved in all promotions, corroborating previous research findings that IBL not only stimulates interest in the topic, but also provides deep knowledge [39]. In fact, quantitative research has shown that IBL boosts student achievement: it improves the acquisition of knowledge and skills and increases students’ desire to learn, making it a more effective strategy for science education than traditional learning [40, 41].

### How did students experience this pedagogical approach?

Although there were some differences between students from the two degree programs, students were satisfied and considered the course and methodology useful. Satisfaction and usefulness are strongly correlated, so differences between students in different degree programs are probably related to the aims of the course (designing and carrying out a research project). Although students were free to choose the research proposal, design, and execution, Human Biology students found it more useful for their future professional life than Medicine students, some of whom do not intend to do research. Furthermore, a stronger correlation was found between usefulness and research skills than usefulness and creativity development, so it could be possible that students perceived more useful the training in research skills for their future professions than the training on creativity. Finally, the performance of the creativity workshop had no effects on the satisfaction with the course and its usefulness, so the general satisfaction with the course and its usefulness could be related to the inquiry model used in this course.

Students were satisfied with their projects and with IBL, remarking that IBL allowed them to learn skills useful for their academic activities and future professions and that the knowledge acquired will be retained. These findings reinforce those of previous studies that found that IBL promotes the development of transversal skills, and domain-specific knowledge, as well as self-reflection, autonomy, taking responsibility for one’s own learning, cooperative work, critical thinking, and long-term knowledge retention [14, 23].

Tutors played an important role in IBL. Students considered that the ideal tutor must have experience as a facilitator, should act as a guide, not only an evaluative figure, and must find the balance between promoting a free environment and redirecting situations when necessary to enhance creativity. This perception agrees with previous publications concluding that facilitators in student-centered approaches should create a safe, free, flexible, open environment to enhance creative thinking [38]. Furthermore, as Savery (2006) explain, educators must guide the learning process and provide thorough debriefing at the conclusion of the learning experience, changing roles from teacher as knowledge provider to tutor as a manager and facilitator of learning [42]. Nevertheless, assessment was an important part of the process of inquiry. Students perceived that formative assessment was useful, but pointed out that evaluation could be subjective depending on the tutors’ and external evaluators’ fields of expertise. Students’ perceptions of assessment are influenced by previous experiences, so students can perceive any intervention involving assessment in various ways, and this can affect their learning process [43].

During this IBL activity, students experienced different emotions. Positive emotions included motivation, engagement, and competitiveness to produce better ideas. Students’ positive emotions are also reflected in their comments about the group environment and creativity workshop. Students considered that positive emotions helped them develop better projects and be more involved. Although some students had negative emotions such as confusion or anxiety about the workload, groups managed to allay most negative emotions. As Litmanen et al. (2012) explained, emotions often depend on the balance between the challenge of the situation and learners’ feelings of competence. Tasks that are too easy or too challenging often result in decreased concentration and involvement. In active learning, students have positive feelings related to motivation and engagement, as well as negative emotions related to anxiety and stress [44]; a good balance enriches learning processes.

### Limitations

#### Design of the study

Some limitations have been found in the design of this study. First, more data are available on the cohorts that conducted the creativity workshop compared to those that did not. This may be because the questionnaire for the 2011–2013 cohorts was delivered in an online format and the number of responses was not as desired. For this reason, since 2014, when the creativity workshop was introduced, the questionnaire was delivered in a classroom and more qualitative data was collected through focus groups and field notes in order to further deepen the usefulness and impact of the workshop. On the other hand, the questionnaire could not be validated, as it is specifically designed for the students who had taken the course and we and we considered that it was not appropriate to test it previously with the same group of students. However, it was discussed in depth with all the researchers and teachers involved in the course.

#### Interpretation of data

The transformation of two non normal distribution variables to normal distributed variables to be coherent with the correlation analysis might have altered a little bit the results and can be considered as a limitation of the interpretation of data. But in fact, the differences have been so minimal that they have not changed the meaning of the results.

In addition, the significant differences identified in the students’ final grades may be influenced by other factors in addition to the creativity workshop. A lower entry scores of the promotions that did not performed the creativity workshop, the students’ profile or the tutors can also be postulated as possible factors involved in these results.

## Conclusions

This study found that students acquired research and creative thinking skills, through an open and interprofessional IBL course. The introduction of stimulatory techniques during the inquiry process has improved the students’ outcomes. In addition, students are highly satisfied with the learning experience and they perceive it as useful for their education. Although restricted to few participants at a single university, some findings of this study suggest that IBL has great potential and can promote skills development. Open-IBL is a promising method for teaching undergraduate students research skills and creativity. Future social challenges require higher cognitive abilities, such as creative and critical thinking, problem-solving, and interdisciplinary collaboration [1, 3, 14] and future research should aim to determine how best to help students develop these abilities.
